# Authors’ Response

**DOI:** 10.1308/rcsann.2014.173a

**Published:** 2014-03

**Authors:** S Mohamad, I Khan, M Shakeel

**Affiliations:** ^1^NHS Tayside, UK; ^2^Nottingham University Hospitals NHS Trust, UK

We read the response by Syed *et al* to our study with interest and are surprised by the conclusions they have drawn from our paper.

It was stated clearly in our article that between 2002 and 2005 the senior author performed dacryocystorhinostomy (DCR) with a stent. As his success rate was lower than comparable evidence, he decided to change his practice in the hope of improving his results and performed DCR without a stent between 2005 and 2006.

Syed *et al*’s queries regarding stents (including removal time) have already been addressed in the methods section of our paper. In the stented group, the stents were removed at three months following surgery. We believe and understand that this is not premature as stent removal can vary from 4 to 24 weeks postoperatively.^1,2^

As for Syed *et al*’s comment on higher subjective success in the non-stented group, it was stated clearly in our publication that the use of stents was associated with eye irritation, displacement of the tube at the medial canthus, nasal crusting and granulation formation at the rhinostomy orifice, which can affect the outcome. This has been supported by the literature in that a stent can be the reason for surgical failure owing to causing granulation tissue formation, synechia formation and punctual erosion.^2–4^

Syed *et al*’s comparison of our study with contradictory evidence in the literature including their own study seems selective. There is clear evidence available in the literature for and against the use of stents in DCR and this was acknowledged in our introduction. Several studies (including a prospective randomised study) show a higher success rate in DCR without stents.^5–9^ Our study concluded that stents are not necessary for primary DCR. This conclusion has been supported by two meta-analyses.^10,11^

The postsaccal blockage for our patients was tested by the ophthalmologists, who used dacryocystography where indicated. This was a very small group of patients and was deemed too insignificant a finding to be elaborated on in our article.

Generally, a retrospective power calculation is not advised. It is not regarded as good practice and if the result of a retrospective study is significant, power is of no interest.^12–14^ It would appear that prospective power has been confused with retrospective power. Depending on how retrospective power is calculated, it might be legitimate to use it to estimate the power and sample size for a future study but it cannot be used legitimately as describing the power of the study from which it is calculated.^15^

## References

[1] Sprekelsen MB, Barberán MT (1996). Endoscopic dacryocystorhinostomy: surgical technique and results. Laryngoscope.

[2] Jin HR, Yeon JY, Choi MY (2006). Endoscopic dacryocystorhinostomy: creation of a large marsupialized lacrimal sac. J Korean Med Sci.

[3] Sharma BR (2008). Non endoscopic endonasal dacryocystorhinostomy versus external dacryocystorhinostomy. Kathmandu Univ Med J.

[4] Zílelíoğlu G, Tekeli O, Uğurba SH (2002). Results of endoscopic endonasal non- laser dacryocystorhinostomy. Doc Ophthalmol.

[5] Smirnov G, Tuomilehto H, Teräsvirta M (2008). Silicone tubing is not necessary after primary endoscopic dacryocystorhinostomy: a prospective randomized study. Am J Rhinol.

[6] Kakkar V, Chugh JP, Sachdeva S (2009). Endoscopic dacryocystorhinostomy with and without silicone stent: a comparative study. Internet J Otorhinolaryngol.

[7] Saeed BM (2012). Endoscopic DCR without stents: clinical guidelines and procedure. Eur Arch Otorhinolaryngol.

[8] Singh M, Jain V, Gupta SC (2004). Intranasal endoscopic DCR (End-DCR) in cases of dacrocystitis. Indian J Otolaryngol Head Neck Surg.

[9] Pittore B, Tan N, Salis G (2010). Endoscopic transnasal dacryocystorhinostomy without stenting: results in 64 consecutive procedures. Acta Otorhinolaryngol Ital.

[10] Feng YF, Cai JQ, Zhang JY, Han XH (2011). A meta-analysis of primary dacryocystorhinostomy with and without silicone intubation. Can J Ophthalmol.

[11] Gu Z, Cao Z (2010). Silicone intubation and endoscopic dacryocystorhinostomy: a meta-analysis. J Otolaryngol Head Neck Surg.

[12] Walters SJ (2009). Consultants’ forum: should post hoc sample size calculations be done?. Pharm Stat.

[13] Hoenig JM, Heisey DM (2001). The abuse of power: the pervasive fallacy of power calculations for data analysis. Am Stat.

[14] Goodman SN, Berlin JA (1994). The use of predicted confidence intervals when planning experiments and the misuse of power when interpreting results. Ann Intern Med.

[15] Common Mistakes Involving Power University of Texas at Austin.

